# Low proviral load in the Kumamoto strain of Japanese Brown cattle infected with the bovine leukemia virus

**DOI:** 10.1186/s12917-023-03738-6

**Published:** 2023-10-02

**Authors:** Toshiaki Inenaga, Koh Fukuoka, Mikiya Sumida, Sakiko Aiba, Kohei Nishikaku, Yuta Matsuno, Tomoko Kobayashi, Kazuhiko Imakawa

**Affiliations:** 1 Laboratory of Animal Management Science, Department of Animal Science, School of Agriculture, Tokai University, Sugido 871-12, Mashiki, Kumamoto, 861-2205 Japan; 2 Research Institute of Agriculture, Tokai University, Toroku 9-1-1, Higashi-ku, Kumamoto, 862-8652 Japan; 3 Kumamoto Prefectural Central Kumamoto Livestock Sanitation Center, Shizume 1666-1, Jonan-machi, Minami-kuKumamoto, 861-4215 Japan; 4 Laboratory of Animal Health, Department of Animal Science, Faculty of Agriculture, Tokyo University of Agriculture, Funako 1737, Atsugi, Kanagawa, 243-0034 Japan; 5 Present Address: National Institute of Health Sciences, Tonomachi 3-25-26, Kawasaki, Kanagawa, 210-9501 Japan; 6 Present Address: Department of Genetics, Albert Einstein College of Medicine, Bronx, N.Y., United States

**Keywords:** BoLA-DRB3, Bovine leukemia virus, Enzootic bovine leukosis, Cattle, Holstein-Friesian, Japanese black, Japanese Brown, Proviral load

## Abstract

**Background:**

The Kumamoto strain of Japanese Brown (JBRK) cattle is a sub-breed of Wagyu and has a different genetic background than that of Japanese Black (JB) cattle. Bovine leukemia virus (BLV) is the pathogen causing enzootic bovine leukosis (EBL), the predominant type of bovine leukosis (BL). EBL is one of the most common bovine infectious diseases in dairy countries, including Japan. Some host genetic factors, including the bovine leukocyte antigen (BoLA)-DRB3 gene, have been associated with the proviral load (PVL) of BLV and/or onset of EBL. Here, we determined the number of BL cases by analyzing prefectural case records in detail. We measured the PVL of BLV-infected JBRK cattle and compared it with that obtained for other major breeds, JB and Holstein-Friesian (HF) cattle. Finally, the relationship between PVL levels and BoLA-DRB3 haplotypes was investigated in BLV-infected JBRK cattle.

**Results:**

We determined the number of BL cases recorded over the past ten years in Kumamoto Prefecture by cattle breed. A limited number of BL cases was observed in JBRK cattle. The proportion of BL cases in the JBRK was lower than that in JB and HF. The PVL was significantly lower in BLV-infected JBRK cattle than that in the JB and HF breeds. Finally, in BLV-infected JBRK cattle, the PVL was not significantly affected by BoLA-DRB3 alleles and haplotypes. BoLA-DRB3 allelic frequency did not differ between BLV-infected JBRK cattle with low PVL and high PVL.

**Conclusions:**

To our knowledge, this is the first report showing that BL occurred less in the JBRK population of Kumamoto Prefecture. After BLV-infection, the PVL was significantly lower in JBRK cattle than that in JB and HF breeds. The genetic factors implicated in maintaining a low PVL have yet to be elucidated, but the BoLA-DRB3 haplotypes are likely not involved.

**Supplementary Information:**

The online version contains supplementary material available at 10.1186/s12917-023-03738-6.

## Background

Japanese Brown cattle is one of the Japanese original beef cattle breeds, which is also known as “Wagyu,” and consists of two strains, Kochi and Kumamoto. Although Japanese Black (JB) cattle are the predominant Wagyu breed and are raised in almost all regions of Japan. In contrast, both strains of Japanese Brown cattle are very less prevalent and raised mostly in their respective districts of origin. The Kumamoto strain of Japanese Brown (JBRK) cattle comprises only 23 thousand heads, most of which are raised in Kumamoto Prefecture, a southern district of Japan. The JBRK cattle originates from Korean native cattle and some European breeds such as Simmental and has different genetic properties than those of JB cattle [1]. For example, JB cattle are characterized by a potential for higher beef marbling [2], whereas JBRK cattle are well-known for their better growth performance [1].

Bovine leukosis (BL) consists of sporadic and enzootic BL (SBL and EBL, respectively). The origin of SBL is unknown; however, EBL is an infectious disease caused by the bovine leukemia virus (BLV), which belongs to the *deltaretrovirus* genus and *Retroviridae* family [3]. In 2021, cases of EBL were officially reported in 19 countries [4], including Japan, and BLV exists with variable prevalence in European, North and South American, and other Asian countries [5, 6]. A nationwide survey was carried out between 2009 and 2011 to measure BLV seroprevalence in 1145 farms distributed across all regions of Japan. It revealed high prevalence, i.e., 40.9% in dairy cattle and 28.7% in beef cattle [7]. Since the number of EBL cases in Japan keeps increasing, from 1365 cases recorded in 2009 to 4334 cases in 2022 (Additional Fig. 1), BLV seroprevalence has likely increased since the latest nationwide survey. Therefore, effective countermeasures are required to decrease the number of BLV-infections and EBL cases.

It is well-known that the disease progression after infection with BLV differs from one cattle to another. A large proportion of seropositive cattle have no symptom, about 30% of infected cattle show persistent lymphocytosis (PL), and less than 5% of infected animals develop malignant lymphoma [8]. The likelihood to exhibit a pathological condition is partly depends on the host’s inheritable genetic factor(s), which may determine the susceptibility or resistance to EBL onset [9, 10]. Among these genetic factors, a polymorphism of the bovine lymphocyte antigen (BoLA)-DRB3 gene, one of bovine major histocompatibility complex genes, has been associated with PL [11, 12] and lymphosarcoma [13]. BoLA-DRB3 alleles and their association with EBL had been extensively studied in many breeds including JB and Holstein-Friesian (HF) cattle. For example, DRB3*09:02, *10:01, and *11:01 alleles are associated with the resistance to lymphoma, whereas DRB3*12:01 and *15:01 alleles have been correlated with a susceptibility to lymphoma in HF cattle [14]. Additionally, the proviral load (PVL) has been linked with some haplotypes of BoLA-DRB3; specifically, DRB3*02:01, *09:02, *14:01:01 and *17:01 alleles are associated with lower PVL and DRB3*12:01 and *1501 alleles are associated with higher PVL in HF cattle [14, 15]. In JB cattle, DRB3*09:02, *10:01 and *11:01 alleles have been linked with lower PVL and DRB3*16:01 allele was related to higher PVL [16, 17]. Recently, an allelic evaluation conducted in both strains of Japanese Brown cattle revealed that the alleles known for their relation to resistance to PL, lymphosarcoma and/or high PVL were not so frequent in JBRK (Additional Table 2). Thus, a different EBL progression is expected to occur in the JBRK cattle, but EBL prevalence in the JBRK cattle has not been reported so far.

The present study classified the recorded BL cases in Kumamoto Prefecture during past ten years referred to the breeds of cattle. Blood samples collected from 1147 cattle at 39 farms in Kumamoto were analyzed to determine the PVL in the JBRK cattle and compare it with that measured in JB and HF cattle. Finally, the relationship between PVL and BoLA-DRB3 haplotypes was investigated in BLV-infected JBRK cattle.

## Results

### Analysis of the numbers of BL cases

**Fig. 1:**
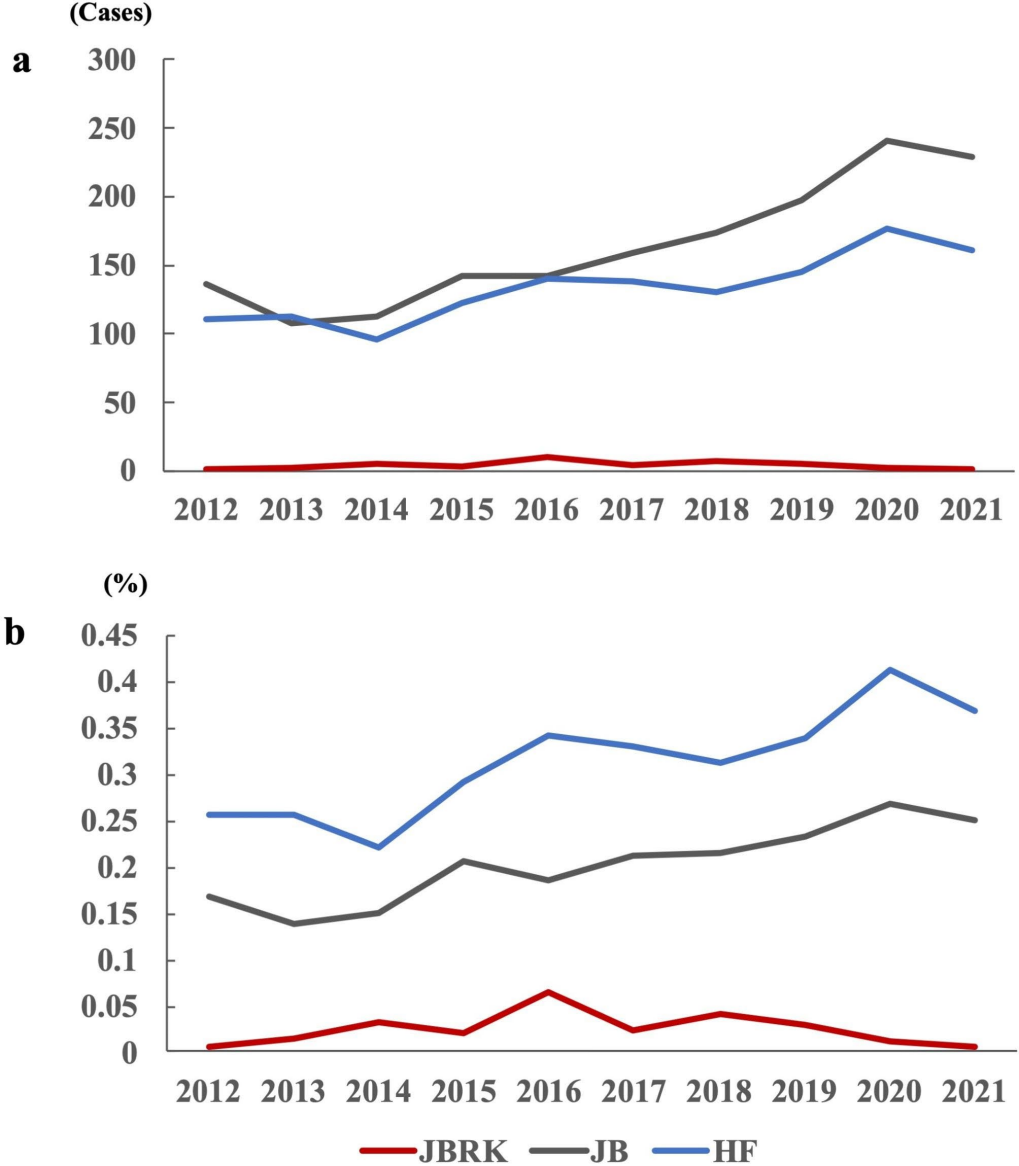
Number of BL cases recorded in Kumamoto Prefecture for each breed (**a**) In Kumamoto Prefecture, the number of reported BL cases in the JBRK cattle was extremely lower than that measured in JB and HF. (**b**) The rate for BL onset, calculated by dividing the number of BL cases by the total number of heads reared in Kumamoto at the same time (described in Additional Table 1), was also lower in the JBRK cattle than that in JB and HF.

The number of BL cases officially reported in Kumamoto Prefecture from 2012 to 2021 was analyzed for each breed. In JB and HF, there were over 100 cases/year for almost all years. On the contrary, there were only 1 or under 10 cases/year in JBRK cattle (Fig. 1a). Additionally, the proportion of BL cases in the JBRK cattle was lower than that in JB and HF raised in Kumamoto Prefecture during the same decade (Fig. 1b) (Additional Table 1).

### PVLs in BLV-infected cattle

The PVL was measured in the whole blood of 1147 cattle (97 JBRK, 672 JB, and 378 HF) confirmed to be positive for anti-BLV antibody. Tables 1 and 2 present the age and PVL of these cattle, respectively. The proportion of elderly cattle was higher in JBRK cattle than that in JB and HF cattle, and JBRK animals were significantly older than other breeds (Table 1, P < 0.00001).

**Table 1:**
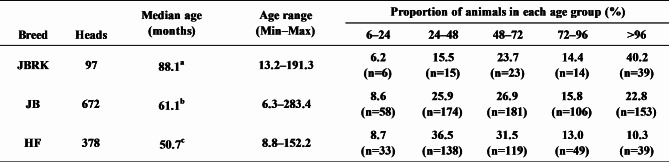
Age distribution in cattle subjected to the PVL study JBRK: Kumamoto strain of Japanese Brown cattle JB: Japanese Black cattle HF: Holstein-Friesian cattle a, b, c: Significantly different among each letter (a-b: P = 0.00000; b-c: P = 0.00010; a-c: P = 0.00000)

**Table 2:**
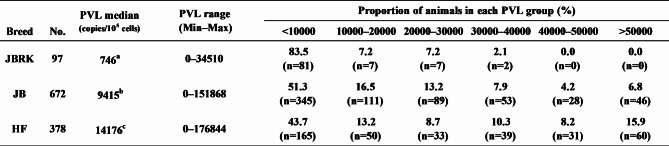
BLV PVL distribution of each breed JBRK: Kumamoto strain of Japanese Brown cattle JB: Japanese Black cattle HF: Holstein-Friesian cattle a, b, c: Significantly different among each letter (a-b: P = 0.00005; b-c: P = 0.00000; a-c: P = 0.00000)

**Fig. 2:**
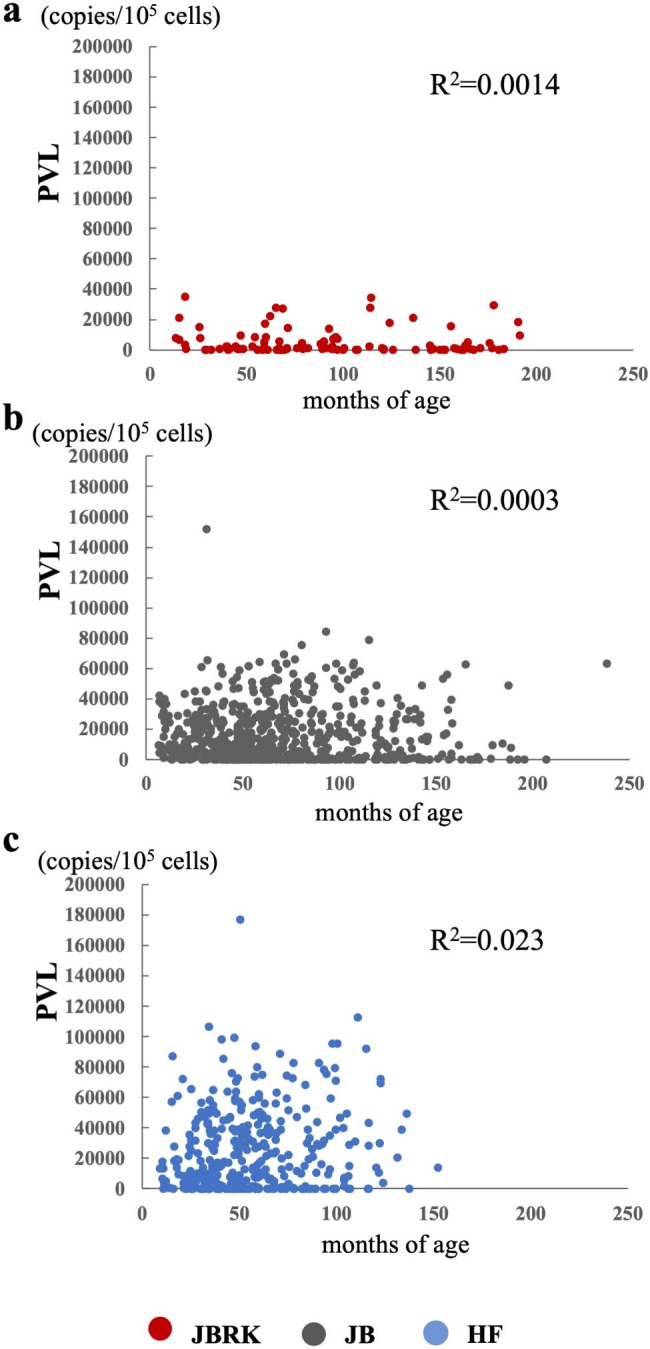
Effects of age on peripheral blood PVL. PVL and age distribution in JBRK (**a**), JB (**b**), and HF (**c**) cattle. There was no correlation between age and PVL levels in each breed

**Fig. 3:**
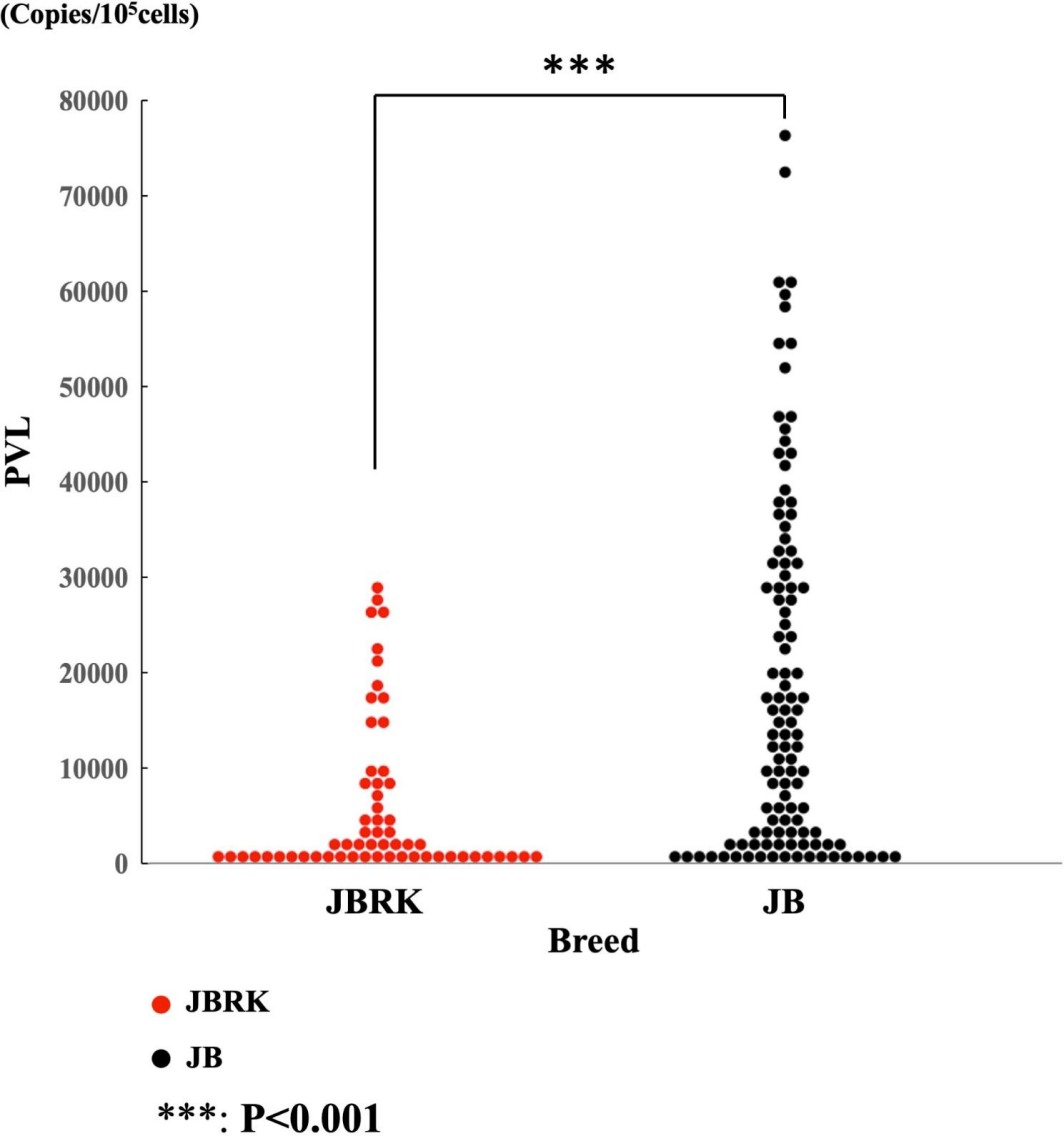
PVL in peripheral blood of 59 JBRK and 111 JB cattle reared together on eight farms The PVL was significantly lower in JBRK cattle than that in JB cattle reared under the same conditions ***: P < 0.001

The PVL was significantly lower in the BLV-infected JBRK cattle than that in JB and HF cattle (Table 2, P<0.00005). PVL of some BLV-infected cattle were under the minimal detection level and their proportions were 10.3% (n = 10) in JBRK, 4.2% (n = 28) in JB, and 2.9% (n = 11) in HF. Since the risk for BLV transmission or EBL onset is related to the PVL [18–20], we categorized the PVL levels into six classes: <10,000, 10,000–20,000, 20,000–30,000, 30,000–40,000, 40,000–50,000, and > 50,000 copies/10^5^ cells. The distribution of these classes in each breed was investigated (Table 2). Over 80% of JBRK cattle had < 10,000 copies/10^5^ cells. In contrast, approximately 50% of JB and HF cattle possessed > 10,000 copies/10^5^ cells. A regression analysis revealed no correlation between the age and PVL in each breed (Fig. 2). To exclude the effects of the rearing environment on proviral increasing, we compared the PVLs of BLV-infected 59 JBRK and 111 JB cattle from 8 farms raising both JBRK and JB cattle (Additional Table 3). The PVL in the JBRK cattle was significantly lower than that in JB cattle raised in the same environments (Fig. 3, P < 0.001).

### Relationship between BoLA-DRB3 haplotypes and the PVL

**Fig. 4:**
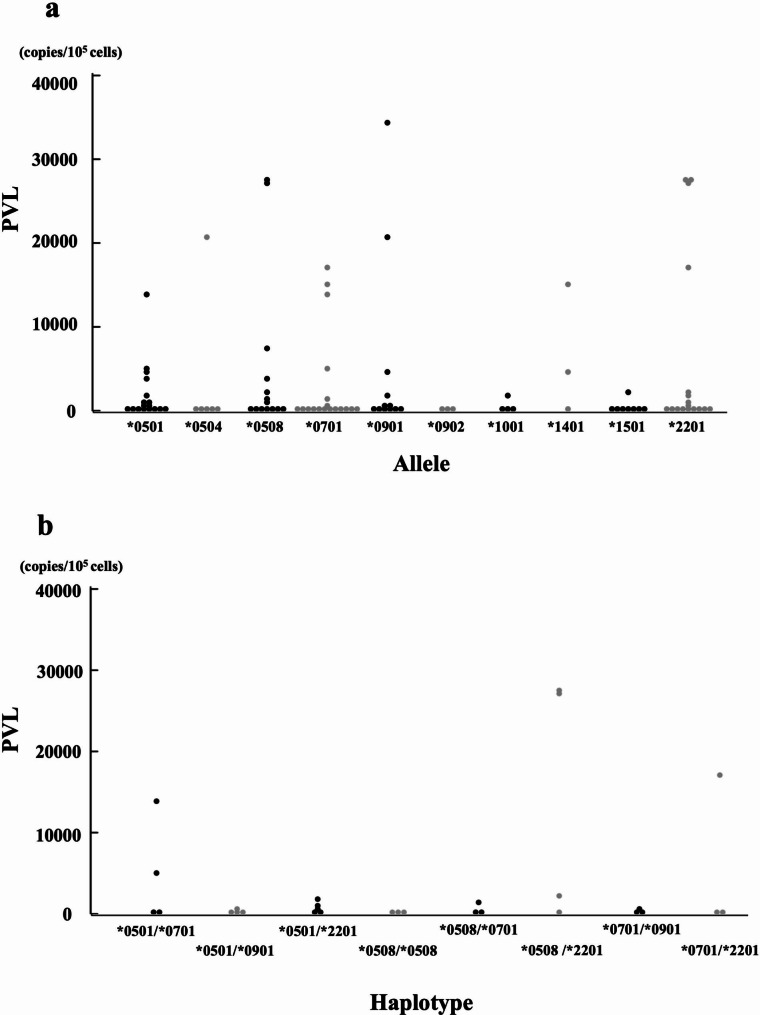
(**a**): Distribution of peripheral blood PVL according to BoLA-DRB3 allele of BLV-infected JBRK cattle. (**b**): Distribution of peripheral blood PVL according to BoLA-DRB3 haplotypes. Black and grey markers were used by turns to distinguish easily. The PVL was not significantly affected by BoLA-DRB3 alleles and haplotypes detected

To study the effects of BoLA-DRB3 haplotypes on the PVL in BLV-infected JBRK cattle, we analyzed the PVL in association with the animal alleles and haplotypes. All detected BoLA-DRB3 alleles and haplotypes were summarized in Additional Tables 2 and 4. To apply statistical analysis, ten alleles and eight haplotypes were selected because those allele and haplotypes were possessed by more than three BLV-infected JBRK cattle. PVL was not significantly affected by the allele and haplotype (Fig. 4, P = 0.08851 and P = 0.7524, respectively). The numbers of alleles possessed by 7 BLV-infected JBRK cattle with > 15,000 copies/10^5^ cells (high proviral load (HPL)) and 38 BLV-infected JBRK cattle with < 1000 copies/10^5^ cells (low priviral load (LPL)) were further analyzed to calculate the odd’s ratios using Fisher’s exact test. Fourteen alleles were found in both of HPL and LPL cattle, and no significant association was found between allele and the PVL (Table 3).

**Table 3:**
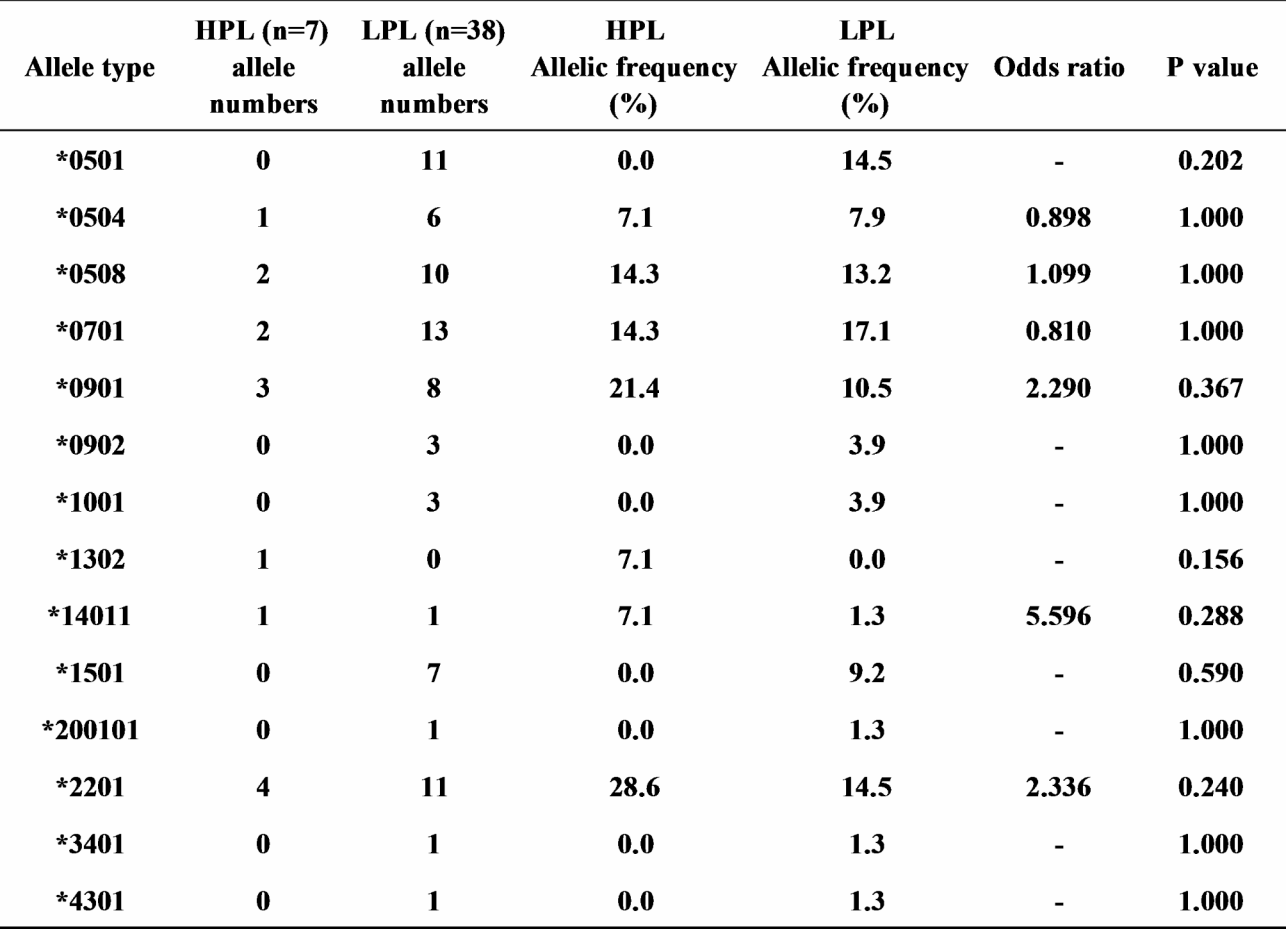
Comparison of detected allelic numbers and frequency between 7 HPL and 38 LPL among BLV- infected JBRK using Fisher’s Exact Test HPL: High proviral load (>15000 copies/10^5^ cells) LPL: Low proviral load (<1000 copies/10^5^ cells) BLV: Bovine leukemia virus JBRK: Kumamoto strain of Japanese Brown cattle

## Discussion

The present study showed that there were fewer BL cases in the JBRK cattle than in JB and HF, even after considering the difference in the rearing numbers. In addition, the PVL in BLV-infected JBRK cattle was significantly lower than that in JB and HF cattle. Moreover, the PVL in the BLV-infected JBRK cattle was lower than that in JB cattle reared in the same environments (same farms). To our knowledge, the present study is the first reporting fewer BL cases and lower PVL in the BLV-infected JBRK cattle than those in JB and HF cattle. Since the PVL reflects the proportion of peripheral lymphocytes with proviral insertion, BLV-infected cattle with a lower PVL have less risk to transmit the BLV to other cattle and to further develop EBL [19, 21]. The very small number of BL cases in JBRK cattle might result from the low PVL. In addition, it could be less risk for transmitting BLV from BLV-infected JBRK cattle than from JB and HF breeds.

About the relationship between the age and PVL of each cattle, no correlation was seen in BLV-infected JBRK cattle as well as other breeds, and these results are consistent with the previous studies [19, 22]. Previous studies have shown a difference in the PVL among breeds of BLV-infected cattle [22, 23]. Additionally, in human T cell leukemia virus type 1 (HTLV-1) infection, of which causative virus is closely related to BLV, ethnic differences in PVL or disease progression have been reported [24, 25]. It has been suggested that these phenomena are due to the host’s genetic diversity, which underlies different immune responses to BLV infection. Korean Hanwoo cattle, one of the ancestral breeds of JBRK, was also known for resistance to BL [26]. There could be common mechanisms for the resistance to EBL in JBRK and Hanwoo cattle. Here, haplotyping and association analyses were conducted using 57 BLV-infected JBRK cattle to confirm the relationship between the resistance to the disease progression and one of candidate factors, the BoLA-DRB3 gene. The PVL of BLV-infected JBRK cattle was not significantly different among animals with different BoLA-DRB3 alleles and haplotypes. Besides, there was no difference in the allelic frequencies between the low PVL and high PVL groups. Therefore, the haplotypes of BoLA-DRB3 might not be linked to PVL in the BLV-infected JBRK cattle. There are other host factors, such as polymorphisms of the p53 gene [27], Spermatogenesis-associated 16 gene [28], tumor necrosis factor-alpha gene [29], and transcription factor ABT1 gene [30], known to affect the PVL. Further investigation including genomics and transcriptomics analyses, is needed to clarify the mechanisms involved in maintaining a low PVL in the BLV-infected JBRK cattle.

## Conclusion

The present study revealed that the BL cases in JBRK was extremely fewer than those in JB and HF. The PVL of BLV-infected JBRK animals was significantly lower than that of JB and HF cattle. This is the first report describing these characteristics of the JBRK cattle. Moreover, the lower PVL in BLV-infected JBRK cattle was not associated with BoLA-DRB3 haplotypes, one of the factors known to affect the PVL level in other breeds. Further analysis is needed to identify the factors implicated in maintaining a low PVL in BLV-infected JBRK cattle.

## Methods

### Quantification of the number of BL cases for each bovine breed

We analyzed prefectural reports on BL cases in detail that have been reported over the the past ten years (2012–2021) to determine the number of BL cases for each breed. The case records were stored in the Livestock Sanitation Centers of Kumamoto Prefecture. These BL cases were diagnosed by veterinarians at farms in Kumamoto and by *post-mortem* inspections at Kumamoto Prefectural Meat Safety Inspection Office. These reports were established in compliance with the Domestic Animal Infectious Diseases Control Law of Japan, and EBL and SBL were grouped as BL. Because the number of heads reared differed among breeds, the number of BL cases was calculated as a proportion of the total number of heads for each breed raised in Kumamoto Prefecture at the same year (Additional Table 1).

### Animals and blood collection

Between April 2019 and March 2021, blood samples were collected from 1810 cattle (JBRK, JB, and HF cattle) of at least six months of age and raised in 39 commercial farms in Kumamoto Prefecture. Because this study was not aimed to survey the positive rate for BLV-antibody, we selected the farms for sampling which were willing to cooperate to our sampling. Among 39 farms, both JBRK and JB cattle were raised together on eight farms. All cattle had no clinical symptoms suggesting BL, and they were subjected to the following tests once between April 2019 and March 2021. The blood samples were collected from the tail vein or jugular vein by well-trained veterinary doctors under holding by a rope. Isolated sera were used for the BLV antibody tests, and anticoagulant-treated whole blood samples were used to measure the PVL and to determine BoLA-DRB3 alleles and haplotypes.

### Measurement of the BLV PVL in the whole blood

The sera were separated from 1810 bloods by centrifuging at 3000 rpm for 10 min, and anti-BLV gp51 antibody levels were measured using a commercial ELISA kit (BLV ELISA kit; Nippon Gene, Toyama, Japan), following the instructions provided by the manufacturer. Among the cattle from which BLV infection had been detected by ELISA, the PVL was measured in the whole blood of 1147 cattle (97 JBRK, 672 JB, and 378 HF). Their genomic DNA was extracted from the anticoagulant-treated blood samples using an automated nucleic acid extraction system (magLEAD 12gc; Precision System Science, Chiba, Japan). To measure the proviral copy numbers, CoCoMo-BLV Primer/Probe (RIKEN GENESIS, Kawasaki, Japan) and THUNDERBIRD Probe qPCR Mix (TOYOBO, Osaka, Japan) were used to prepare a reaction mix and a quantitative polymerase chain reaction (PCR) was run on a StepOne real-time PCR system (Thermo Fisher Scientific, Waltham, MA, USA). The whole blood PVLs were calculated from the threshold cycle (Ct) numbers and the calibration curves using BLV Plasmid DNA/Dilution Solution (RIKEN GENESIS). PVL represented the copy numbers per 10^5^ cells and 0 copy was attributed to samples with undetectable PVL level.

### BoLA-DRB3 allelic analysis

To investigate the relationship between the whole blood PVL and the alleles of BoLA-DRB3 in BLV-infected JBRK cattle, haplotyping was performed by analyzing DNA sequencing data as described previously [31]. Genomic DNA was extracted from anti-coagulant-treated peripheral whole blood samples collected from 57 BLV-infected JBRK cattle using a commercial kit (QIAmp DNA mini kit, QIAGEN, Hilden, Germany). The exon 2 domain of BoLA-DRB3 was amplified from approximately 100ng of the extracted DNA by PCR using 2 primers (DRB3FRW: 5’-CGC TCC TGT GAY CAG ATC TAT CC-3’ and DRB3REV: 5’-CAC CCC CGC GCT CAC C-3’) and PrimeSTAR GXL DNA Polymerase (Takara Bio, Shiga, Japan). The following cycling conditions were used: 98 °C for 2 min, followed by 35 cycles of denaturation at 98 °C for 10 s, annealing at 63 °C for 15 s and extension at 68 C for 10 s. The PCR products were purified using the illustra ExoProStar kit (Cytiva, Tokyo, Japan) and directly sequenced (FASMAC, Kanagawa, Japan). The obtained sequences were assembled and analyzed using SequencherTM5.2.4 (GeneCodes, Michigan, United States) and manually compared with the reference BoLA-DRB3 sequence registered in the IPD-MHC database (https://www.ebi.ac.uk/ipd/mhc/group/BoLA/).

### Statistical analysis

All statistical analyses were conducted using R software version 4.1.2. The PVL and age of each cattle, estimated using the Shapiro–Wilk test, were not distributed normally. Thus, nonparametric analyses were performed using PVL or age as an object variable and breeds as a fixed effect. If a factor had a significant effect, post hoc multicomparison tests were conducted using Steel–Dwass test. Correlations between the PVL and ages of cattle were examined by regression analysis. The relationship between the haplotypes of BoLA-DRB3 and PVL was analyzed using the nonparametric Kruskal–Wallis test and the risk for higher PVL was analyzed using Fisher’s exact test. The level of significance was set at 5%.

### Electronic supplementary material

Below is the link to the electronic supplementary material.

Supplementary Material 1

Supplementary Material 2

Supplementary Material 3

Supplementary Material 4

Supplementary Material 5

Supplementary Material 6

## List of abbreviations

BL: Bovine leukosis
BLV: Bovine leukemia virus
BoLA: Bovine leukocyte antigen
EBL: Enzootic bovine leukosis
ELISA: Enzyme-linked immunosorbent assay
HF: Holstein-Friesian
JB: Japanese Black
JBRK: Kumamoto strain of Japanese Brown
PCR: Polymerase chain reaction
PL: Persistent lymphocytosis
PVL: Proviral load
SBL: Sporadic bovine leukosis
