# Supplementary figures and images for: Low proviral load in the Kumamoto strain of Japanese Brown cattle infected with the bovine leukemia virus
Source: BMC Vet Res. 2023 Oct 2;19:185. doi: 10.1186/s12917-023-03738-6 (PMC10544446; doi:10.1186/s12917-023-03738-6)

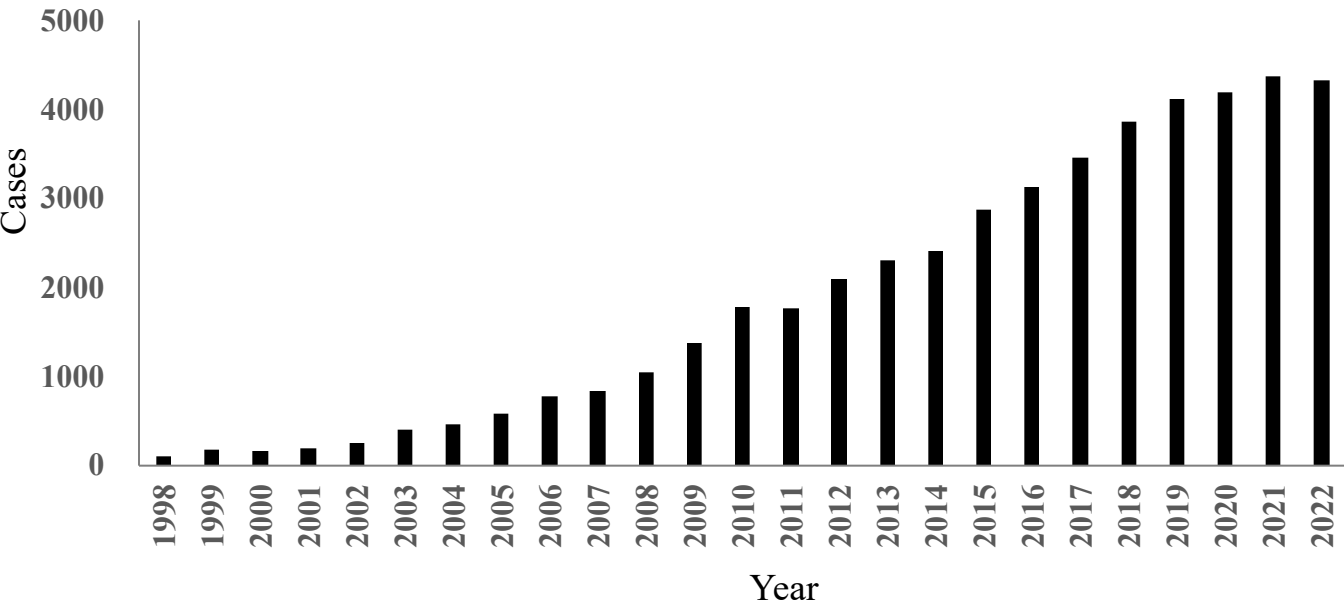

Supplement: Supplementary file 1 — Supplementary Material 1 [file 12917_2023_3738_MOESM1_ESM.pdf]
