# Supplementary material for: Low proviral load in the Kumamoto strain of Japanese Brown cattle infected with the bovine leukemia virus
Source: BMC Vet Res. 2023 Oct 2;19:185. doi: 10.1186/s12917-023-03738-6 (PMC10544446; doi:10.1186/s12917-023-03738-6)
Supplement: Supplementary file 2 — Supplementary Material 2 [file 12917_2023_3738_MOESM2_ESM.pdf]

Additional table 1.
Frequency of all detected BoLA-DRB3 allele among 57 JBRK.

Allele	*0501	*0504	*0508	*0701	*0702	*0901	*0902	*1001	*1302	*14011	*1501	*200101	*20012	*2201	*3401	*4301
**Heads**	**17**	**6**	**14**	**18**	**1**	**12**	**3**	**4**	**1**	**3**	**8**	**2**	**1**	**17**	**1**	**1**
**Allelic frequency(%)**	**14.9**	**5.3**	**12.3**	**15.8**	**0.9**	**10.5**	**2.6**	**3.5**	**0.9**	**2.6**	**7.0**	**1.8**	**0.9**	**14.9**	**0.9**	**0.9**
**Susceptibility in other breeds (Breeds)**							**R (HF, JB)**	**R (JB)**		**R (HF)**	**S (HF)**					
**References**							[14, 15, 16]	[17]		[14]	[15]					

R: Resistant to high PVL
S: Susceptible to high PVL
HF: Holstein-Friesian cattle
JB: Japanese Black cattle
