# Supplementary material for: Low proviral load in the Kumamoto strain of Japanese Brown cattle infected with the bovine leukemia virus
Source: BMC Vet Res. 2023 Oct 2;19:185. doi: 10.1186/s12917-023-03738-6 (PMC10544446; doi:10.1186/s12917-023-03738-6)
Supplement: Supplementary file 3 — Supplementary Material 3 [file 12917_2023_3738_MOESM3_ESM.pdf]

Additional table 2.
Total heads of each breed rearing in Kumamoto Prefecture in 2012-2021 reported in Kumamoto Stock Raising Statistics by Kumamoto Prefectural Office.

	Heads		
**Year**	**JBRK**	**JB**	**HF**
**2012**	**14202**	**81137**	**43216**
**2013**	**15086**	**77881**	**44037**
**2014**	**15049**	**74952**	**43297**
**2015**	**14968**	**68716**	**41621**
**2016**	**15183**	**76319**	**41000**
**2017**	**16843**	**75053**	**41793**
**2018**	**17163**	**80291**	**41595**
**2019**	**16245**	**84171**	**42553**
**2020**	**16970**	**89397**	**42842**
**2021**	**16483**	**90949**	**43537**

JBRK: Kumamoto strain of Japanese Brown cattle
JB: Japanese Black cattle
HF: Holstein-Friesian cattle
