# Supplementary material for: Low proviral load in the Kumamoto strain of Japanese Brown cattle infected with the bovine leukemia virus
Source: BMC Vet Res. 2023 Oct 2;19:185. doi: 10.1186/s12917-023-03738-6 (PMC10544446; doi:10.1186/s12917-023-03738-6)
Supplement: Supplementary file 4 — Supplementary Material 4 [file 12917_2023_3738_MOESM4_ESM.pdf]

Additional table 3.
Distribution of numbers and PVL of cattle from 8 farms raising both JBRK and JB cattle together.

Farm		A		B		C		D		E		F		G		H	
Breed		JBRK	JB	JBRK	JB	JBRK	JB	JBRK	JB	JBRK	JB	JBRK	JB	JBRK	JB	JBRK	JB
Number (Heads)		17	5	4	16	4	17	4	2	8	10	12	48	9	9	1	4
PVL (copies/10^5 cells)	Range (Min-Max)	0-28967	253-32103	16-16960	65-60839	0-12	0-44983	12-361	237-5351	6-27568	48-53988	0-17522	10-63511	268-22167	0-78943	-	0-56045
	Median	1919	3115	1133	4498	7	7662	211	2794	194	21897	1390	17938	9464	12382	136	9506

JBRK: Kumamoto strain of Japanese Brown cattle
JB: Japanese Black cattle
PVL: Proviral load
