# Supplementary material for: Low proviral load in the Kumamoto strain of Japanese Brown cattle infected with the bovine leukemia virus
Source: BMC Vet Res. 2023 Oct 2;19:185. doi: 10.1186/s12917-023-03738-6 (PMC10544446; doi:10.1186/s12917-023-03738-6)
Supplement: Supplementary file 5 — Supplementary Material 5 [file 12917_2023_3738_MOESM5_ESM.pdf]

Additional table 4.
Frequency of all detected BoLA-DRB3 haplotypes among 57 JBRK.

Haplotypes	*0501/0504	*0501/*0508	*0501/*0701	*0501/*0901	*0501/*200101	*0501/*2201	*0504/*0504	*0504/*0901	*0504/*0902	*0504/*14011	*0508/*0508	*0508/*0701	*0508/*1501	*0508/*20012	*0508/*2201	*0701/*0901
Heads	1	2	4	4	1	5	1	1	2	1	3	3	1	1	4	3
Haplotype frequency(%)	1.8	3.5	7.0	7.0	1.8	8.8	1.8	1.8	3.5	1.8	5.3	5.3	1.8	1.8	7.0	5.3

Haplotypes	*0701/*0902	*0701/*1001	*0701/*14011	*0701/*1501	*0701/*2201	*0702/*1501	*0901/*0901	*0901/*1001	*0901/*14011	*1001/*2201	*1302/*2201	*1501/*200101	*1501/*2201	*1501/*4301	*2201/*3401
Heads	1	1	1	2	3	1	1	2	1	1	1	1	2	1	1
Haplotype frequency(%)	1.8	1.8	1.8	3.5	5.3	1.8	1.8	3.5	1.8	1.8	1.8	1.8	3.5	1.8	1.8

BoLA: Bovine leukocyte antigen
JBRK: Kumamoto strain of Japanese Brown cattle
