# Supplementary material for: Low proviral load in the Kumamoto strain of Japanese Brown cattle infected with the bovine leukemia virus
Source: BMC Vet Res. 2023 Oct 2;19:185. doi: 10.1186/s12917-023-03738-6 (PMC10544446; doi:10.1186/s12917-023-03738-6)
Supplement: Supplementary file 6 — Supplementary Material 6 [file 12917_2023_3738_MOESM6_ESM.docx]

**Additional files**

Additional figure 1

.pdf

Additional figure 1

Numbers of bovine leukosis cases reported in Japan from 1998 to 2022. (From “Annual statistics of notifiable animal infectious diseases” reported by Ministry of Agriculture, Forestry and Fisheries of Japan)

URL: https://www.maff.go.jp/j/syouan/douei/kansi_densen/attach/pdf/kansi_densen-63.pdf

Additional table 1

.pdf

Additional table 1

Frequency of all detected BoLA-DRB3 allele among 57 JBRK.

R: Resistant to high PVL

S: Susceptible to high PVL

HF: Holstein-Friesian cattle

JB: Japanese Black cattle

Additional table 2

.pdf

Additional table 2

Total heads of each breed rearing in Kumamoto Prefecture in 2012-2021 reported in Kumamoto Stock Raising Statistics by Kumamoto Prefectural Office.

JBRK: Kumamoto strain of Japanese Brown cattle

JB: Japanese Black cattle

HF: Holstein-Friesian cattle

Additional table 3

.pdf

Additional table 3

Distribution of numbers and PVL of cattle from 8 farms raising both JBRK and JB cattle together.

JBRK: Kumamoto strain of Japanese Brown cattle

JB: Japanese Black cattle

PVL: Proviral load

Additional table 4

.pdf

Additional table 4

Frequency of all detected BoLA-DRB3 haplotypes among 57 JBRK.

BoLA: Bovine leukocyte antigen

JBRK: Kumamoto strain of Japanese Brown cattle
